# Letter from Paris

**Published:** 1867-10

**Authors:** John D'Oyley Evans


### CORRESPONDENCE.

### *Letter from Paris.*

PARIS, *July* 31st, 1867.

MESSRS. EDITORS:—

IN a paper addressed to the Academy of Paris by Dr. Rosenthal of Ulm, a very important and perhaps a useful circumstance is brought to light.

Dr. Rosenthal having effected some experiments on a poison brought over from Malacca, and exercising an elective action on the heart, discovered that it acts with less intensity on fowls.

As this poison contains a large proportion of strychnine, he resumed his experiments with that poison in a *pure state;* its characteristic property, as you are well aware being that of producing tetanus.

Our author thus succeeded in determining the quantities of strychnine requisite to produce death in different animals. The doses do not differ considerably, though they are not absolutely the same. The ingestion of the poison was always effected by the mouth, and in the shape of a watery solution.

Rabbits require a milligm. of nitrate of strychnine for every 500 gms. of their weight; guinea-pigs, sparrows, and pigeons will absorb double that quantity before they die ; chickens, on the contrary, will bear nearly twelve times that proportion. On this occasion, Dr. Rosenthal observed that by establishing artificial breathing in rabbits, so as to suppress all natural motion on respiration, they might be made to bear much larger doses of the poison than in the normal state. Under such artificial action the animal will walk on the table as if it had taken no poison; but no sooner is artificial respiration suspended than convulsions will begin again very rapidly and worse than ever. If artificial breathing be again applied, the fits will cease, and the animal return to its normal state. Hence it appears that a poison may be dormant in the blood of an animal, without producing its effect. And yet the poison has not lost its power, since it can resume it as soon as the artificial action is suspended. This shows that an abundance of oxygen in the blood will paralyze the effects of the poison, which thereby may be so completely neutralised as to save the animal's life.

This may be done by continuing artificial respiration for three or four hours, during which time the poison is eliminated or more probably decomposed and converted into an innocuous substance.

These experiments may be found to be of use in cases of traumatic lockjaw; and it is not unreasonable to predict that patients of that class, whose life is now despaired of, may be saved by introducing oxygen into their organism by means of artificial respiration.

I have not yet had time to visit the Exhibition, where several medals, I have heard, have been awarded to Americans for superiority in manufacturing mineral teeth.

John D'Oyley Evans, D D.S.

